# Intraindividual long term stability and response corridors of cytokines in healthy volunteers detected by a standardized whole-blood culture system for bed-side application

**DOI:** 10.1186/1471-2288-12-112

**Published:** 2012-08-01

**Authors:** Silke C Mueller, Reinhard März, Manfred Schmolz, Bernd Drewelow

**Affiliations:** 1 Institute for Clinical Pharmacology, Medical Faculty, University of Rostock, Schillingallee 70, 18057, Rostock, Germany; 2 Peter-Hannweg-Str. 8, 90768 Fürth, and Ohm-University of Applied Sciences Nuremberg, Nuremberg, Germany; 3 EDI (Experimental & Diagnostic Immunology) GmbH, Aspenhaustr. 25, 72770, Reutlingen, Germany

## Abstract

**Background:**

The variation of immune cell activities over time is an immanent property of the human immune system, as can be measured by the stimulated secretion of cytokines in cell cultures. However, inter-individual variability is considerably higher. Especially the latter is the major reason why it has not been possible to establish international standard values for cytokines as was possible for other parameters, such as leukocyte sub-population numbers. In this trial, a highly standardized whole-blood culture model (TrueCulture®), developed to characterise drug effects on cells of the human immune system in clinical trials, was used to analyse cytokine patterns in the blood samples of 12 healthy subjects over a period of one month.

**Methods:**

After an overnight fast, 12 healthy subjects donated blood three times a week on three consecutive days over a period of 4 weeks. TruCulture® blood collection and whole-blood culture systems were used to measure whole-blood leukocyte stimulation. The levels of IL-2, IL-5, IL-13, IL-6, IL-8, IL-10, IFNγ, and MCP-1 in the culture supernatants were quantified by sandwich ELISA.

**Results:**

The pattern of cytokine concentrations in the supernatants of the stimulated whole-blood cultures was highly individual, but considerably stable over the whole observation period of 4 weeks.

**Conclusions:**

By using TruCulture® it seems feasible to determine subject-specific cytokine reference patterns, for example under healthy conditions, or before starting an experimental treatment, e.g. during a clinical trial, against which changes in the behaviour of the immune system can be detected more accurately in future.

## Background

Leukocytes respond to various types of signals, such as infectious agents [1], tumour cells [2], dying cells [3], allergens [4], stress hormones [5], but also changes in the composition of the intestinal microbiota [6], and – last, but not least – drug activities [7]. This diversity of daily challenges makes it hard to find a truly resting immune system in humans at all. On the other hand, each subject represents a highly complex genetic combination of polymorphisms [8], making its immune system a virtually unique selection of high and low responder states of the genes encoding for example most of the chemokines and cytokines that regulate immune responses and inflammatory processes.

Together, this necessarily leads to enormous inter-individual variability regarding cytokine levels in plasma, serum, or culture supernatants, thereby preventing the defining of internationally accepted standard values for cytokines, chemokines, or other mediators related to immune cell activation. As a consequence, except for inbred animals kept germ-free, it is almost impossible to precisely define a basal level of immune cell activity as being “normal” (one might even argue that particularly the immune system of a germ-free animal will not even respond to activation in a “normal = healthy” way). Immune cell function can be measured by various methods, all of which usually employ multistep techniques, before the cells are allowed to develop their activities [9]. Each single step in these procedures creates its own variability and will be done in a slightly different way in each lab in the world. The type of cell culture media, stimulants, plastic materials (like culture plates), etc. may also differ from lab to lab and can easily lead to differences in the results. This makes it highly problematic to compare results from different groups and different trials [10].

Another issue when comparing results from leukocyte activity assays pertains to the fact the blood is often stored for different periods (often ranging from as short as a few minutes up to 24 h, or longer) before the preparation of the white blood cells began. As with any other type of primary cell, also leukocytes suffer from a loss of activities over time upon storage. Again, results of immune cell activation assays using leukocytes entering the tests at such different condition cannot be compared reliably. Even within the same clinical study the shipping time of the blood to the cell culture lab may vary widely from trial centre to trial centre.

Thus, PBMC (peripheral blood mononuclear cell) cultures, although being the most widely used cell culture method to characterise leukocytes at a functional level, are considered the one with the most built-in variables [9].

Far less problematic in that regard are whole-blood cultures (WBC), because they eliminate most of the manipulations necessary to set up PBMC cultures. In addition, cell adherence is significantly reduced due to the fact that the leukocytes do not sediment directly onto the plastic surface (where they become adherent and thus artificially activated), but onto a bed of red blood cells (forming the “buffy coat”). This prevents cell stress and thus, presumably, the variability of the results. Another advantage, which was recognized quite early in the use of whole-blood cultures, was that the activities seen in WBCs resemble the cell activities *in vivo* much better [11]. Numerous whole-blood test systems have been published to date and this technique can be regarded as being widely accepted meanwhile.

Nevertheless, one of the major issues still existed so far: the usually ill-defined period between the blood draw and the beginning of the cell culture. It would clearly be advantageous to eliminate all type of storage and shipment which could impair the quality of the cells to be examined and to start the culture within a few minutes after drawing the blood. However, this is only possible when the blood donation can be performed directly in the cell culture lab. This is rarely the case in clinical trials and virtually never in field research. Clinical trial sites usually not only lack personnel experienced in cell culture techniques, but also any type of regular culture lab equipment (sterile hoods, CO_2_ incubators, etc.).

These considerations eventually lead to the design of an on-site whole-blood culture, performed in a peculiar type of syringe, as well as a proprietary culture medium, allowing the culture of leukoyctes without needing a CO_2_ incubator. By means of these medium-filled syringes (“TruCulture® blood collection and whole-blood culture system”) it is possible to start the activated culture of the leukocytes contained in the whole-blood at the very moment when the blood enters the syringe. The plunger of this syringe can be broken away easily after the blood draw has finished. This leaves a closed tube, which then is immediately incubated in a dry block thermostat at 37°C. This procedure eliminates any need to store and ship the blood, so the cell culture starts without delay and virtually manipulation-free. Most important, especially for clinical trials, this maximizes the standardisation of such functional assays around the human immune system.

In order to achieve a physiological, balanced type of activation involving cells of the native as well as of the adaptive compartments of the immune system, a combination of 3 different stimuli was used:

a) Zymosan, which is a β-glucan from yeast cells; this forms phagocytable particles, which bind to TLR2/TLR6 [12], CD11/18 [13], and Dectin [14], that not only are ingested by monocytes and granulocytes, but also trigger the release of cytokines and chemokines [15]

b) *Staphylococcus* enterotoxin B, a potent stimulator of mainly Th1 lymphocytes [16]

c) anti-CD28 antibodies, an antibody binding to an important co-stimulating receptor, which – together with SEB antibodies – gives an additional shift towards Th2 cells [17].

The cell culture endpoints chosen for this experimental study consisted of a selection of cytokines and chemokines relevant to judge inflammatory processes. IL-2 and IFNγ both pertain to the Th1-type of immune response [18], while IL-5 and IL-13 rather mark the Th2-driven reactions [19]. These two T helper cell types are seen mutual counter-regulators [20]. Under the given conditions, IL-6 and IL-10 are mainly produced by the cells of the native immune system (as a product of zymosan stimulation, [15]) and also can be seen mediators that have opposite effects in the activation of immune cells (IL-6 being mostly of pro-inflammatory activity [21], while one of the major functions of IL-10 is its inhibitory effect on cytokine synthesis [22]. IL-8 and MCP-1 belong to the chemokines and can be seen the most prominent representatives of the both major sub-classes, the α- (or CXC) and the β-chemokines (CC), [23].

## Methods

### Volunteers and study design

Fifteen healthy male, non-smoking volunteers with a mean age of 26.3 ± 3.8 years, height of 172.6 ± 6.1 cm, weight of 72.5 ± 10.2 kg and a body mass index of 22.3 ± 2.6 kg/m^2^ were enrolled into the study. Participants were determined to be healthy on the basis of medical history, physical examination, electrocardiography, and routine urine, clinical chemistry and hematologic screening including parameters of inflammation such as C-reactive protein, protein electrophoresis and erythrocyte sedimentation rate. Furthermore, all volunteers were required to have no laboratory evidence of hepatitis B, hepatitis C or human immunodeficiency virus infection or drug abuse. Participants were excluded if they had any relevant medical history, a history of acute infection within the last 4 weeks before the start of the study or during the study, received active vaccination within 4 weeks before the study start, use of any prescription or over the counter drug (including herbal medicine) within 4 weeks before enrolment into the study, had a surgical procedure or injury within 12 weeks before the start of the study or donated erythrocytes or thrombocytes within 4 weeks before the start of the study. Extreme physical activity was not allowed during the study.

After an overnight fast a blood sample was drawn between 8:00 AM and 9:00 AM on Mondays, Tuesdays and Wednesdays of four consecutive weeks. The TruCulture® blood collection and whole-blood culture device was used according to the instructions of the manufacturer. Volunteers that missed more than 1 scheduled blood sample or developed an infection were excluded from this evaluation. 3 drop-outs occurred during this 4 week period.

The study was approved by the Ethics Committee of the University of Rostock.

### TruCulture® blood collection and whole-blood culture system

The TruCulture® blood collection and whole-blood culture device (Myriad RBM, Inc., Austin, USA) was operated as described in the instruction manual. The TruCulture® syringe-tubes, containing a proprietary nutrient solution together with a combination of stimulants, were sent to the trial site at −40°C on dry ice and stored at −20°C at the trial site. The stimulants used in this trial were zymosan, a ß-glucan from yeast (Sigma-Aldrich, Deisenhofen, Germany), used at a final concentration of 300 μg/mL, staphylococcus enterotoxin B (SEB, from the Bernhard Nocht Institute, Hamburg, Germany; final conc. 200 ng/mL), as well as anti-CD28 antibodies (Beckmann-Coulter, Frankfurt, Germany, final conc. 1 μg/mL). This combination of stimuli was used to activate simultaneously the phagocytes (granulocytes and monocytes) by zymosan, as well as the T-cells by SEB + anti-CD28, giving a very physiological pattern of activation. The TruCulture® syringe front end is a screw cap into which a male-type connector with a rubber septum is inserted. Together with a special butterfly needle system (21 G) attached to a female-type connector that punctures this rubber septum during the blood draw (Sarstedt, Nümbrecht, Germany), this ensures a closed state of these syringe-tubes, before as well as after the blood draw, thereby preventing the contamination of the cultures.

Half an hour prior to the blood draw these tubes are thawed at ambient temperature. Then empty syringe-tubes are connected to the butterfly needle system, the cubital vein of the volunteer is punctured and the tubing system of the butterfly needle is filled completely with blood (to ensure that just blood and no air is drawn into the TruCulture® tubes). The reason for this is that, for the sake of standardisation, the TruCulture® syringe-tubes are equipped with a spacer attached to the plunger, enabling the user to precisely draw 1 mL of blood into the syringe-tubes, pre-filled with 2 mL of culture medium.

After filling the butterfly tubing with blood the empty syringe-tube is replaced by the TruCulture® tube to be filled and exactly 1 mL of blood is drawn into the tube. Thereafter, the TruCulture® is taken off the connector and its plunger is broken away. This leaves a simple screw-cap tube, which is immediately put into a desktop dry-block incubator (VLM Vogler, Bielefeld, Germany), where it is incubated for 24 h.

At the end of this incubation period the tubes are taken out of the block again and the screw cap is opened. Then a special valve is inserted, which separates the sedimented cells from the supernatants containing the cytokines and chemokines to be measured. The valve shuts off the supernatant within a second, so the secretion of any more mediators can be stopped immediately. This enables an easy standardisation of the culture period. After inserting the valve the tube is frozen at −20°C until measuring the cytokine concentrations.

### Mediator quantification

Cytokine and chemokine concentrations in the supernatants were determined by sandwich-type enzyme-linked immune-sorbent assays (ELISAs). These were performed using the matched antibody pairs from R&D Systems together with the cytokine standards from R&D Systems, according to the procedures suggested by the manufacturer.

### Statistical methods

For the analysis of the intraindividual variation over the 4 weeks period, the data of the 3 consecutive days of blood sampling at 8:00 AM every week were subsumed under week [1-4] and plotted for every subject separately as dots; also, the overall mean for each subject over 4 Weeks was calculated along with its 95-% confidence interval to allow significance judgements. Additionally, an explorative discriminance analysis was conducted to identify subjects with similar cytokine patterns.

## Results

The immune cells of each blood donor were challenged with a standardised stimulus (zymosan + SEB + anti-CD28) in whole-blood cultures, which had been developed for the peculiar situation of clinical trials (TruCulture®). All cultures of each of the donors tested in this study showed sufficient responses to the stimuli used and complete sets of results were available for each of the study subjects.

**Figure 1:**
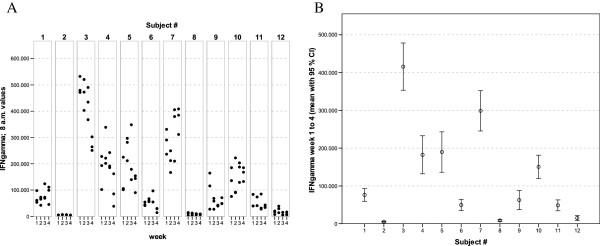
** IFNγ values of all 12 healthy donors of 3 consecutive days per week over a 4 week period (A).** Part **(B)** shows the mean of all 12 measurements for each of the donors together with its 95% confidence interval.

**Figure 2:**
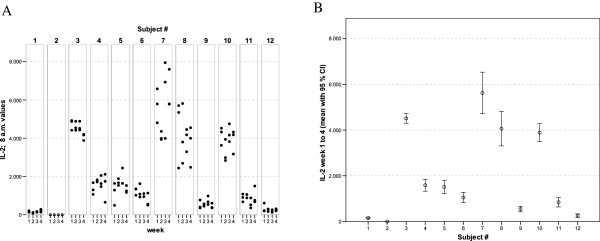
** IL-2 values of all 12 healthy donors of 3 consecutive days per week over a 4 week period (A).** Part **(B)** shows the mean of all 12 measurements for each of the donors together with its 95% confidence interval.

**Figure 3:**
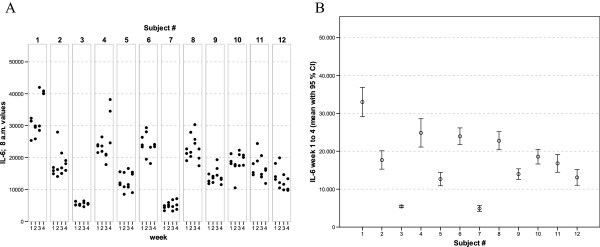
** IL-6 values of all 12 healthy donors of 3 consecutive days per week over a 4 week period (A).** Part **(B) **shows the mean of all 12 measurements for each of the donors together with its 95% confidence interval.

**Figure 4:**
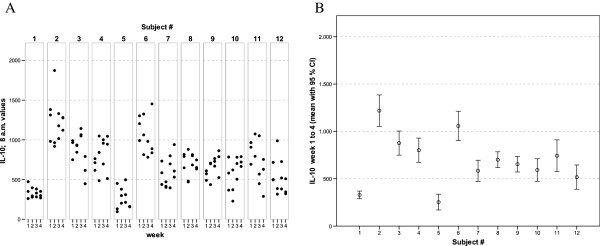
** IL-10 values of all 12 healthy donors of 3 consecutive days per week over a 4 week period (A).** Part **(B)** shows the mean of all 12 measurements for each of the donors together with its 95% confidence interval.

**Figure 5:**
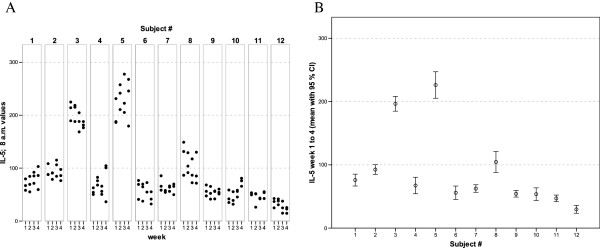
** IL-5 values of all 12 healthy donors of 3 consecutive days per week over a 4 week period (A).** Part **(B)** shows the mean of all 12 measurements for each of the donors together with its 95% confidence interval.

**Figure 6:**
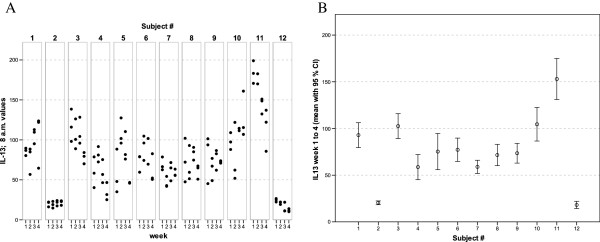
** IL-13 values of all 12 healthy donors of 3 consecutive days per week over a 4 week period (A).** Part **(B)** shows the mean of all 12 measurements for each of the donors together with its 95% confidence interval.

**Figure 7:**
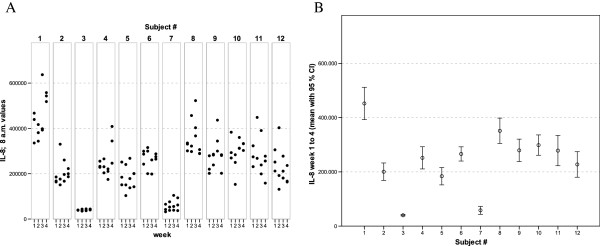
** IL-8 values of all 12 healthy donors of 3 consecutive days per week over a 4 week period (A).** Part **(B)** shows the mean of all 12 measurements for each of the donors together with its 95% confidence interval.

**Figure 8:**
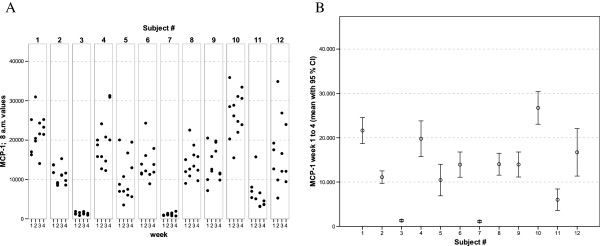
** MCP-1 values of all 12 healthy donors of 3 consecutive days per week over a 4 week period (A).** Part **(B)** shows the mean of all 12 measurements for each of the donors together with its 95% confidence interval.

With regards to the mediator levels measured in these whole-blood culture supernatants, virtually each of the cytokines and chemokines showed a more or less subject-specific and relatively stable average concentration level throughout the whole observation period. In general, low, medium and high responses to the standardised experimental stimulation could be observed (see Figures 1, 2, 3, 4, 5, 6, 7, 8).

The widest inter-individual range of variation of all mediators measured was observed for the cytokine IFNγ (Figure 1), a mediator typically secreted by Th1 lymphocytes, especially when peripheral blood leukocytes are activated by SEB. Volunteer #3 for example, demonstrated a very high T cell-related activity in his cultures concerning the concentrations measured for IFNγ, IL-2, IL-5, as well as IL-13 (see Figures 1, 2, 5, and 6). On the other hand, despite exhibiting the highest IFNγ value of all subjects, the leukocytes of the same donor (#3) secreted the lowest concentrations of the two chemokines in this set of mediators, IL-8 and MCP-1 (Figures 7 and 8), and similar was true for IL-6 (Figure 3).

Completely different profiles were observed for other subjects, such as #8, whose concentrations of IL-2 were among the highest values measured in this study, while only negligible amounts of IFNγ were detectable in his culture supernatants (Figures 1 and 2). Most other mediators were detected at intermediate concentrations in the samples of this donor, except for IL-8, for which the second highest mean value of all measured concentrations was found. Thus, each individual obviously displays its “personal ranges” of response when looking at the single cytokines.

Taking together all eight mediators measured in this series of experiments, it was possible for each of the trial subjects to establish a fingerprint-like cytokine pattern, which showed considerable stability over the whole observation period, as can be derived from the mean values plus confidence intervals shown in Figures 1, 2, 3, 4, 5, 6, 7, 8.

**Figure 9:**
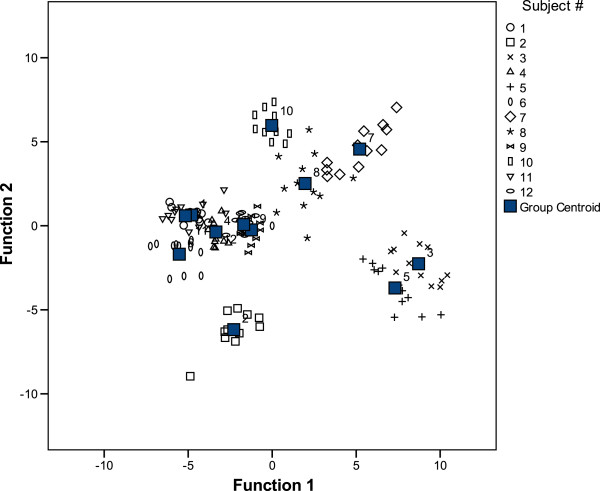
**Explorative discriminance analysis.** The data of the 8 cytokines on 3 consecutive days from the 12 subjects over 4 weeks were subjected to an exploratory discriminance analysis in order to identify groups with similar cytokine patterns among the individuals. Remarkably, individuals do not change their relative position among the others during the 4 weeks period.

The discriminance analysis was conducted on an exploratory level only, as the case number was small. It confirmed that the whole cytokine pattern of a subject is stable over time, and that, clearly, some subjects are more similar in their pattern than others. Among the 12 subjects of the trial, the presence of four “groups” with separate sub-groups (see Figure 9) could be identified:

Group A: consisting of subjects ## 1, 4, 6, 9, 11, and 12;

Group B: subjects # 5 and # 3;

as well as

Group C: with subjects ## 7, 8, and 10, forming a more loosely grouped set of subjects, obviously sharing some common features in their cytokine patterns that suffice to differentiate these from the rest of the study population, after all;

“Group D”: subject # 2, eventually, seems to differ in his profile from all other donors and may therefore belong to a separate group of individuals.

## Discussion

Functional tests using leukocyte cultures are one of the most reliable ways to objectively measure drug effects on the human immune system. However, especially those using isolated leukocytes do have a couple of drawbacks, among which are that they are usually laborious and depend on the availability of not only experienced personnel, but also special lab equipment. In addition, their performance often varies, depending mostly on differences in the handling of the cells, but also on the type of plastic materials or the source and the purity of media supplements used.

Most of these disadvantages can be eliminated by using whole-blood cultures, which do not only avoid those manipulation steps that are the most stressful for the immune cells (centrifugation and re-suspension), but also ensure a rapid transfer of the cells from the blood circulation to the culture, thereby avoiding larger and sustained shifts in the ambient temperature, which may also lead to changes in cellular activities. Whole-blood cultures were also recognized to more precisely reflect the effects of drugs on the human immune system than those using isolated leukocytes [10].

Another obstacle in the characterisation of pharmacological effects on cells of the human immune system is the well-known inter-individual variability in leukocyte activities [17], [18]. Together with the non-existence of international standard values for cytokines this makes it very difficult in clinical trials to differentiate drug-dependent effects from random changes in immune cell function.

The goal of the investigation presented here was therefore to analyze the variations of several cytokines over a longer period under controlled conditions regarding life-style, food intake and day-time. It turned out that, in the sample, the inter-individual range of responses was very large. However, when looking at the subject level, there was a remarkable signal stability over the 4 weeks of observation in our study and the responses of each subject took place in a relatively small corridor, which differed between the certain individuals significantly (Figures 1,2,3,4,5,6,7,8). Both findings were unexpected in their explicitness, although similar observations were described earlier, using regular cell culture techniques still requiring traditional cell culture lab equipment [24,25].

The results obtained in this study clearly confirmed the results of others in the way, that there are in fact considerable differences to be found between the stimulated cultures of the 12 donors tested in this trial. However, our trial now suggests that there is a remarkable consistency underlying this inter-individual variability: The cytokine levels in activated cultures of each single donor do in fact change only little, provided (a) the donor stays healthy, (b) the stimulus used to activate the immune cells does not change, and (c) a cell culture system is used that reduces external influences on leukocyte activities due to manipulation artifacts.

A major part of the inter-individual variation in immune cell activities is most probably caused by genetic factors. Despite the fact that the cytokine or chemokines genes are virtually the same in all human beings, numerous single nucleotide polymorphisms (SNPs) have been characterised in the past decade, which often influence mediator gene expression levels to a certain degree [19]. To date, more than 20,000,000 SNPs are listed for human genes in one of the largest SNP databases [26], with not less than 25 entries just for IL-2. Each one of these SNPs does or does not have an impact on the concentration of the protein the gene codes for. Moreover, also activating receptors do show their own polymorphisms and thus contribute to the inter-individual variability in response to stimulation (e.g., for human TLR2, one of the receptors of zymosan, 64 SNPs are listed in the NCBI SNP database, [27]).

Therefore, it is self-evident that any human being will form a mosaic of SNPs that decides about the potency of his/her immune cells to create a certain pattern of protein mediators upon a well-defined stimulation. In order to be able to detect such a pattern in a reproducible way, a test system is required that does not increase the degree of variability due to its technical complexity. From the low degree of variability observed in our current investigation it seems to be justified to consider TruCulture® a tool that makes it possible to collect reliable results even in the absence of regular cell culture facilities or experienced personnel. This is due to the high degree of standardisation regarding the manufacturing process of these syringe-tubes, as well as the restriction of the culture preparation steps to the basic necessities (i.e.: the blood draw).

The use of such culture systems enables the clinical researchers to determine individual baseline patterns and differentiate these clearly from the changes observed later during disease or after the onset of experimental or standard therapies. The more mediators, soluble receptors and other activation markers will be included in these profiles, the higher the probability to discover a meaningful spectrum of changes in leukocyte activity and the higher the safety to not miss important signal. It is conceivable that for each state of a disease or type of drug characteristic patterns may occur, which even allow to calculate specific scores making it easier to interpret the results for daily clinical use.

## Conclusions

A newly developed, standardised bed-site whole-blood leukocyte culture system was used to determine the consistency over time of complex cytokine patterns in healthy volunteers. Despite the well-known inter-individual variation in mediator synthesis, the homogeneity of intra-individual cytokine patterns which was found in this study does have the potential to serve as subject-specific reference values not only in health and disease, but also in clinical trials.

## Competing interests

Reinhard März: I declare that I have not received any reimbursement, fees, funding, salaries from the organisation that may gain or lose from the publication, and I do not hold stocks or shares of this organisation, and I am not engaged in any patent business relating to the content of the manuscript.

Manfred Schmolz is the CEO of EDI GmbH, a CRO, which invented the TruCulture® blood collection and whole-blood culture system.

Silke Mueller: I declare that I have not received any reimbursement, fees, funding, salaries from the organisation that may gain or lose from the publication, and I do not hold stocks or shares of this organisation, and I am not engaged in any patent business relating to the content of the manuscript.

Bernd Drewelow: I declare that I have not received any reimbursement, fees, funding, salaries from the organisation that may gain or lose from the publication, and I do not hold stocks or shares of this organisation, and I am not engaged in any patent business relating to the content of the manuscript.

## Authors’ contributions

SM and BD were conducting this clinical trial and wrote the major parts of this manuscript. RM performed the analysis of the data and contributed to the writing of the related parts of this manuscript. MS was involved in writing the TruCulture®-specific sections of the manuscript. All authors read and approved the final manuscript.

## Pre-publication history

The pre-publication history for this paper can be accessed here:

http://www.biomedcentral.com/1471-2288/12/112/prepub
